# OsSIDP301, a Member of the DUF1644 Family, Negatively Regulates Salt Stress and Grain Size in Rice

**DOI:** 10.3389/fpls.2022.863233

**Published:** 2022-07-28

**Authors:** Li Ge, Hongming Guo, Xiu Li, Ming Tang, Chiming Guo, Han Bao, Linjuan Huang, Yin Yi, Yuchao Cui, Liang Chen

**Affiliations:** ^1^Xiamen Key Laboratory for Plant Genetics, School of Life Sciences, Xiamen University, Xiamen, China; ^2^Key Laboratory of National Forestry and Grassland Administration on Biodiversity Conservation in Karst Mountainous Areas of Southwestern China, School of Life Sciences, Guizhou Normal University, Guiyang, China; ^3^Fujian Key Laboratory of Subtropical Plant Physiology and Biochemistry, Fujian Institute of Subtropical Botany, Xiamen, China

**Keywords:** OsSIDP301, salt stress, ABA signaling, grain size, cell expansion

## Abstract

As a major environmental factor, salt stress substantially retards growth and reduces the productivity of rice (*Oryza sativa*). Members of the DUF1644 family, “the domains of unknown function 1644 motif” are predicted to play an essential regulatory role in response to abiotic stress. However, the specific molecular mechanisms of most members of this family remain elusive. Here, we report that the *OsSIDP301* (stress-induced DUF1644 protein) was induced by salt stress and abscisic acid (ABA). We found that overexpression of *OsSIDP301* (OE) in plants conferred salt hypersensitivity and reduced grain size, whereas plants with *OsSIDP301* RNA interference (RNAi) exhibited salt tolerance and increased grain size in rice. *OsSIDP301* determines salt stress tolerance by modulating genes involved in the salt-response and ABA signaling pathways. Further studies suggest that *OsSIDP301* regulates grain size by influencing cell expansion in spikelet hulls. Moreover, OsSIDP301 interacts with OsBUL1 COMPLEX1 (OsBC1), which positively regulates grain size in rice. Our findings reveal that *OsSIDP301* functions as a negative regulator of salt stress and grain size, and repressing its expression represents a promising strategy for improving salt stress tolerance and yield in rice.

## Introduction

Adverse environmental conditions limiting agricultural productivity and threatening security, such as saline alkalization of soil, severely affect plant development and damage one-fifth of the irrigated land (Rasool et al., 2013; Roy et al., 2014; Zhu, 2016; Morton et al., 2019). Rice (*Oryza sativa*), which is one of the most important cereal crops, is sensitive to saline alkalinization (Hussain et al., 2017; Chang et al., 2019). To meet the demand for food for a rapidly growing population and to compensate for the ever-reducing availability of arable land, it is necessary to urgently develop salt-tolerant rice varieties through efficient molecular breeding technologies (Godfray et al., 2010; Qian et al., 2016). Extensive studies have demonstrated that salt stress usually causes ion toxicity (e.g., Na^+^ and Cl^–^), hyperosmotic stress, and secondary stress (e.g., oxidative damage; Zhu, 2002). Plants have evolved various strategies to cope with salt stress, such as the accumulation of antioxidants, osmolytes, phytohormones, and salt-tolerant plant growth-promoting rhizobacteria (Singh et al., 2022; Sinha et al., 2022), which are involved in an intricate signaling network (Zhao et al., 2020). First, the signals are triggered by sodium ion sensing and a cytosolic Ca^2+^ concentration increase, which depend on salt stress signaling sensors, namely, glycosyl inositol phosphoryl ceramides, and diverse Ca^2+^-dependent proteins (Jiang et al., 2019). Subsequently, the salt sensitivity signaling pathway is activated to exclude Na^+^ and activate downstream salt stress-responsive genes (Yang and Guo, 2018). Recent studies on rice have shown that multiple ion transporters, such as *HIGH-AFFINITY K^+^ TRANSPORTER 1* (*HKT1*) (Wei et al., 2021), *HKT4* (Wang et al., 2015), *HKT8* (Hauser and Horie, 2010), *HIGH-AFFINITY POTASSIUM* (*K*^+^) *TRANSPORTER 1* (*OsHAK1*) (Chen et al., 2015), and *OsHAK21* (Shen et al., 2015), are involved in regulating the homeostasis of Na^+^/K^+^ and play a positive role in salt stress management.

Rice yield is determined by the number of effective tillers, number of grains per panicle, and grain weight. Moreover, the grain length, width, and thickness directly influence grain weight. In recent decades, numerous quantitative trait loci and genes that regulate grain size have been identified, such as *GRAIN WEIGHT 2* (Song et al., 2007), *BIG GRAIN 1* (Liu et al., 2015), *RICE SQUAMOSA PROMOTER-BINDING-LIKE 13* (Si et al., 2016), and *GRAIN SIZE and ABIOTIC STRESS TOLERANCE 1* (GSA1; Dong et al., 2020). Various studies indicated that the regulation of grain size is associated with multiple signaling factors, including phytohormones, protein kinases, and transcription factors (Zuo and Li, 2014; Li et al., 2019). Notably, brassinosteroid (BR) has been highlighted in connection with grain shape (Li et al., 2019), and mutants with *dwarf2* and *dwarf11* (involved in BR biosynthesis) displayed a typical BR-deficient phenotype and have small and short grains (Hong et al., 2005; Tanabe et al., 2005; Fang et al., 2016; Wu et al., 2016). The BRASSINOSTEROID UPREGULATED 1 (BU1), a putative helix–loop–helix (HLH) transcription factor, displays typical BR phenotypes and enlarged grains, and also functions as a brassinosteroid UPREGULATED-LIKE 1 (BUL1) and OsBC1 (Tanaka et al., 2009; Jang et al., 2017).

To date, although *EARLY FLOWERING 4a* (OsELF4a; Wang et al., 2021) and *PSEUDO-RESPONSE REGULATOR 73* (OsPRR73; Wei et al., 2021) were reported to positively regulate salt tolerance and grain yield by modulating downstream genes and related proteins, and *GSA1* positively regulates salt tolerance and grain size by modulating metabolic flux redirection (Dong et al., 2020), the synergistic regulatory network between abiotic stress and grain yield remains largely unknown (Dong et al., 2020).

Domains of unknown function 1644 (DUF1644) is a large group of proteins (Bateman et al., 2010). Although *OsSIDP366* and *OsSIDP361* have been reported to be involved in abiotic stress (Guo et al., 2016; Li et al., 2016), the coordinated regulatory network between salt tolerance and grain size remains unclear. In this study, to explore the function of *OsSIDP301*, which encodes a DUF1644 protein, we developed plants with gene overexpression (OE), gene silencing (RNA interference; RNAi), and gene knock-out. To better understand the role of *OsSIDP301* in plant growth and development, we screened for OsSIDP301 interacting proteins, and OsBC1, a basic HLH transcription factor, was identified. In addition, gene silencing plants of *OsBC1* also showed a smaller grain size in rice. Our findings revealed that *OsSIDP301* was involved in synergistic regulation of salt stress tolerance and grain size, and may have a direct application value in rice.

## Materials and Methods

### Plant Materials and Trait Measurements

In this study, rice (japonica variety TaiPei309, TP309) plants were cultivated with an interplant spacing of 20 cm × 20 cm in the field at Xiamen University, Fujian Province, China, under natural growing conditions. The grain length, width, thickness, and 1,000-grain weight were measured using filled grains after maturation using a Vernier caliper. TP309 was used as the background for the genetic transformation and control of physiological experiments.

### Generation of Transgenic Lines

*OsSIDP301* was amplified from TP309 and cloned into the pCXUN-Flag (Chen et al., 2009) vector to generate the overexpression line p*Ubi*: *OsSIDP301* (*OsSIDP301*^OE^). Knockdown and knockout plants were generated using RNAi technology and clustered regularly interspaced short palindromic repeats (CRISPR/Cas9) technology. *OsSIDP301* gene-specific RNA sequences and guide sequences were cloned into the pH7GWIWGII vector (*OsSIDP301*^RNAi^) and pH-Ubi-cas9 vector (*OsSIDP301*^cas9^). For the p*OsSIDP301*: glucuronidase (*GUS*) vector, a DNA fragment containing the *OsSIDP301* promoter was inserted into the pCXGUS-P vector (Chen et al., 2009). These constructs were introduced into TP309 *via Agrobacterium*-mediated transformation. The primers used are listed in Supplementary Table 1.

### β-Glucuronidase Staining Analysis

To localize the transcripts of *OsSIDP301* in TP309 tissue, a 988-bp sequence upstream of the start codon was cloned into the pCXGUS-P vector, and the construct pro*OsSIDP301*:*GUS* was introduced into TP309 by *Agrobacterium tumefaciens* with the EHA105-mediated transformation method. The activity of GUS in transgenic plants was detected using a histochemistry assay, as previously described (Jefferson, 1987). The panicles and spikelets of different developmental stages were soaked in a staining solution (50 mM phosphate buffer saline at pH = 7.2, 2 mM potassium ferricyanide, 2 mM potassium ferrocyanide, 0.2% Triton-X-100, and 1 mg/ml X-Gluc) at 37 °C in an incubator with constant temperature. The materials were washed in 70% ethanol after 24 h and images were taken using a stereoscope.

### Subcellular Localization of OsSIDP301

To investigate the subcellular localization of the OsSIDP301 protein, the coding sequence of *OsSIDP301* was fused with a green fluorescent protein (GFP) cloned into the plasmid of pXDG (Chen et al., 2009) by using ligation-independent cloning technology. Then the constructs of pXDG-OsSIDP301, membrane localization maker (Scamp-mCherry), and nuclear localization maker (NLS-RFP) were co-expressed in tobacco leaves or rice protoplast. The GFP fluorescence signal was observed with a laser scanning confocal microscope (Zeiss LSM, Germany).

### Stress Treatments

For phenotype analysis after germination growth, rice seeds of the WT and transgenic lines were sterilized with 70% ethanol for 1 min and then soaked in sodium hypochlorite solution [containing 0.8% (W/V) effective chlorine] for 20 min. After washing with sterile distilled water, the seeds were first germinated on standard 1/2 MS or selection solid medium (1/2 MS + hygromycin) and then transferred to 1/2 MS medium with or without NaCl (100 mM and 150 mM) or abscisic acid (ABA) (3 and 5 μM). After 1 week of growth in the incubator with a photoperiod of 12 h light (30 °C)/12 h dark (25 °C), the shoot length, root length, fresh weight, and dry weight were measured. Two-week-old seedlings grown on 1/2 MS medium were treated with liquid NaCl (150 mM) for 5 days and then recovered in 1/2 MS solution. Survival rates were counted after 7–30 days, and the criterion for death was the absence of the green shoots.

### Physiology Parameter Characterization

The total chlorophyll content was detected following a previously described method (Zhou et al., 2018). Briefly, the leaf samples (100 mg) of 3-week-old seedlings were soaked and sealed in 4 ml of 95% (W/V) ethanol and then placed in an incubator at 37 °C in the dark for 2 weeks. The soaking liquid was centrifuged at 8,000 *g*, and the supernatant absorbance (*A*) was read at 665, 649, and 470 nm, respectively.

The malondialdehyde (MDA) content was measured following a previously described method (Du et al., 2018a). Briefly, the leaf samples (20 mg) of 3-week-old seedlings were homogenized in 5 ml of 10% (W/V) trichloroacetic acid and centrifuged at 2,000 *g* for 10 min at 4 °C. The supernatant was reacted with an equal volume 0.67% (W/V) thiobarbituric acid and then boiled for 15 min and centrifuged at 5,000 *g*. The *A* of the supernatant was read at 450, 532, and 600 nm, respectively.

The proline concentration was measured following a previously described method (Du et al., 2018b). Leaf samples (100–200 mg) of 3-week-old seedlings were soaked in 5 ml of 3% (W/V) sulfosalicylic acid in 10-ml tubes and boiled for 30 min, and then a reaction mixture was prepared using the supernatant, 2.5% (W/V) ninhydrin reagent, and glacial acetic acid in equal volume and boiled for 30 min. The reaction mixture was added to a double toluene solution of glacial acetic acid and reacted for 24 h. The *A* of the supernatant was read at 520 nm.

The Na^+^ content was measured using the previously described method (Fang et al., 2018). Briefly, the leaves were oven-dried at 65 °C for 10 days, 100 mg samples were extracted in 20 ml of 1 M HCl at 25 °C, and the supernatant was filtered and diluted. Finally, Na^+^ was quantified using flame atomic absorption spectrophotometry (FP6410).

The activity of antioxidant enzyme catalase (CAT) was determined based on the rate of decomposition of H_2_O_2_ as described protocols with the reagent test kit of Nanjing jiancheng (A007-1-1). The leaves (100 mg) were homogenized in 50 mM phosphate buffer sodium (pH = 7.8), centrifuged (1,000 *g*, 20 min, 4 °C), and then the supernatant was used for the analysis of CAT activity.

The diaminobenzidine (DAB) staining assays were performed as described previously (You et al., 2018).

### RNA Isolation and Quantitative Real-Time PCR

Total RNA was extracted using an RNA extraction kit (Promega, Shanghai, China). Subsequently, RNA (2–4 μg) was reverse-transcribed into complementary DNA with a reverse transcription kit (Promega). Quantitative real-time PCR (RT-qPCR) was performed using gene-specific primers (Supplementary Table 1) and a detection system on a ROCHE LightCycler 96 instrument (Switzerland). Samples from the wild-type or unstressed wild-type tissue were selected as controls, the gene for *OsACTINI* was detected in parallel and used as an internal reference, and the data were analyzed using the comparative Ct method. Each experiment was performed in triplicates. The primers used are listed in Supplementary Table 1.

### RNA-Seq

For Illumina sequencing, 2.5–3 cm panicle and 20-day-old leaves of TP309 were collected with three biological replicates and RNA was isolated for library preparation using Illumina sequencing by Novogene Bioinformatics Technology Co., Ltd., Beijing, China. RNA-seq reads were aligned to rice cultivar NP reference genome and gene information.^1^

### Yeast Two-Hybrid Library

A Y187 yeast cDNA library of young seedlings was constructed using the pGADT7 vector (TaKaRa Bio, Dalian, China). The coding sequence of *OsSIDP301* was introduced into the pGBKT7 vector and transformed into Y2H-Gold yeast cells to screen the cDNA library. Library screening was performed using the mating method, according to TaKaRa Bio. Putative interaction proteins were characterized by sequencing the positive colonies. The coding region of the putative gene was cloned into PGADT7 and co-transformed with PGBKT7-OsSIDP301 to confirm the interaction in the Y2H-Gold strain. The positive control (PGBKT7-53 + PGADT7-T) was used as previously described by using the Y2H system. Primers used are listed in Supplementary Table 1.

### Bimolecular Fluorescence Complementation Analysis

*OsSIDP301* was cloned into the *Pac*I/*Asc*I restriction sites of the 2YN [pSAT6-n(1–174)EYFP-C1] and 2YC (pSAT6-cEYFP-C1-B vector) to generate constructs for the bimolecular fluorescence complementation (BiFC), respectively (Citovsky et al., 2006). For transient expression, *A. tumefaciens* strain Gv3101 carrying the combined constructs (*OsSIDP301*^YC^ + *OsSIDP301*^YN^) was co-expressed with the P19 strains into leaves of 1-month-old *Nicotiana benthamiana*. The YFP fluorescence signal was examined using a Zeiss laser scanning confocal microscope at 48–72 h after infiltration. Primers used are listed in Supplementary Table 1.

### Statistical Analysis

Each experiment contained three biological replicates. All data analyses were performed using GraphPad Prism version 7.0.0 for Windows (GraphPad Software, San Diego, CA, United States^2^). Statistical analysis was performed using IBM SPSS Statistics for Windows (version 25.0; IBM Corp., Armonk, NY, United States), and means were compared by computing unpaired Student’s *t*-tests. Pairwise multiple parameter comparisons were made using Duncan’s multiple range test to obtain the significance groups (Duncan, 1955).

## Results

### Expression Analysis and Subcellular Localization of OsSIDP301

**FIGURE 1:**
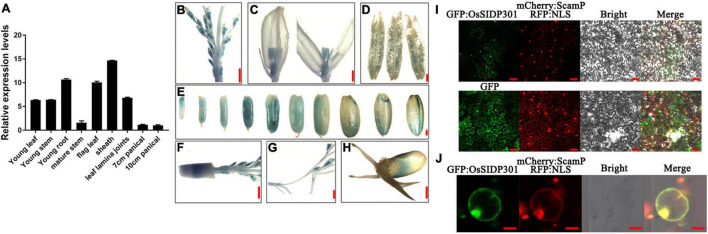
Expression profiling and subcellular localization. **(A)** Transcript levels of *OsSIDP301* in various tissues. *OsACTIN1* was used normalized and data are shown as mean ± SD (*n* = 3). Promoter–GUS activity of *OsSIDP301* in young panicle **(B,F,G)**, pollen **(D)**, stigma **(C)**, seed maturation stage, **(E)** and germination stage **(H)**, bar = 1 mm **(B,C,E–H)**, bar = 10 μm **(D)**. Subcellular localization of OsSIDP301-GFP in *N. benthamiana* leaves **(I)**, bar = 50 μm and rice protoplast **(J)**, bar = 20 μm.

To investigate the expression pattern of *OsSIDP301*, real-time quantitative PCR (RT-qPCR) assay was performed. *OsSIDP301* was expressed in all tissues of rice, including sheath, leaf lamina joints, and panicle (Figure 1A). To further explore the expression pattern of *OsSIDP301* in more detail, transgenic plants harboring the β-glucuronidase (GUS) reporter were generated under the control of the *OsSIDP301* promoter, and GUS staining showed that *OsSIDP301* was highly expressed in stamen, pistil, and grain (Figures 1B–H). To analyze the protein subcellar localization of OsSIDP301, the GFP-OsSIDP301 fused protein was constructed and introduced into the *N. benthamiana* leaves and rice protoplasts. Confocal results showed that GFP signals can be co-localized with membrane localization signal (SCAMP1-mCherry) and nuclear localization signal (NLS-RFP) (Figures 1I,J). Therefore, OsSIDP301 was localized in both the cell membrane and nucleus.

### *OsSIDP301* Knockdown Confers Salt Tolerance in Rice

**FIGURE 2:**
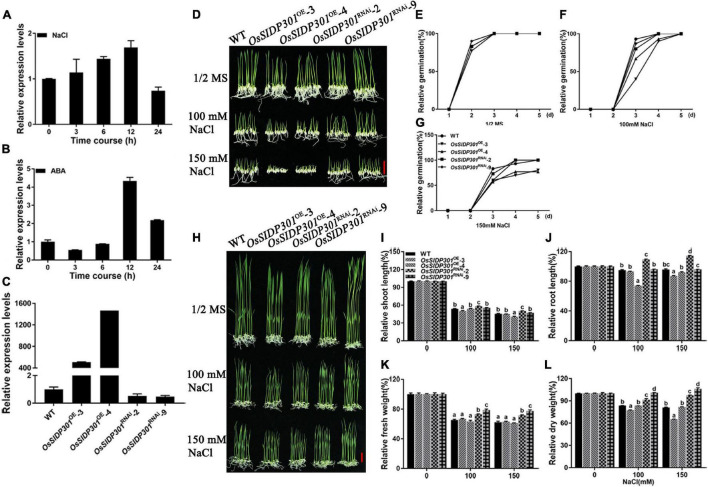
Phenotype of *OsSIDP301* transgenic plants with NaCl treatment. Transcript levels of *OsSIDP301* were induced by 100 mM NaCl **(A)** and 100 μM ABA **(B)** in 3-week-old seedlings. **(C)** Relative expression levels of *OsSIDP301* between WT, *OsSIDP301*^OE^, and *OsSIDP301*^RNAi^ plants. **(D)** Phenotype of *OsSIDP301* transgenic plants with NaCl treatment at germination stage, bar = 2 cm. Comparisons of germination rate between WT, *OsSIDP301*^OE^, and *OsSIDP301^RNAi^* plants under normal condition **(E)** and with NaCl treatment for 7 days **(F,G)**. **(H)** Phenotype of *OsSIDP301* transgenic plants with NaCl treatment at the seedling stage, bar = 2 cm. Comparisons of shoot length **(I)**, root length **(J)**, fresh weight **(K)**, and dry weight **(L)** between WT, *OsSIDP301*^OE^, and *OsSIDP301*^RNAi^ plants with or without NaCl treatment for 7 days. Data are shown as mean ± SD (*n* = 8), different letters suggest significant difference at *P* < 0.05.

DUF1644 members have been reported to play important roles in abiotic stress. For example, *OsSIDP366* and *OsSIDP361* are involved in salt and drought stress (Guo et al., 2016; Li et al., 2016). To determine whether *OsSIDP301* responds to abiotic stress, *OsSIDP301* was first detected in leaves treated with NaCl or ABA. *OsSIDP301* expression was induced by salt stress and ABA treatment (Figures 2A,B). Subsequently, RNAi technology was used to generate *OsSIDP301* knockdown transgenic lines (referred to as *OsSIDP301*^RNAi^), and *OsSIDP301* was driven by a maize (*Zea mays*) *ubiquitin* promoter to obtain its overexpression lines (referred to as *OsSIDP301*^OE^), both in the TP309 background. Two lines of each transgenic plant were selected for the study and their expressions were confirmed by RT-qPCR (Figure 2C).

**FIGURE 3:**
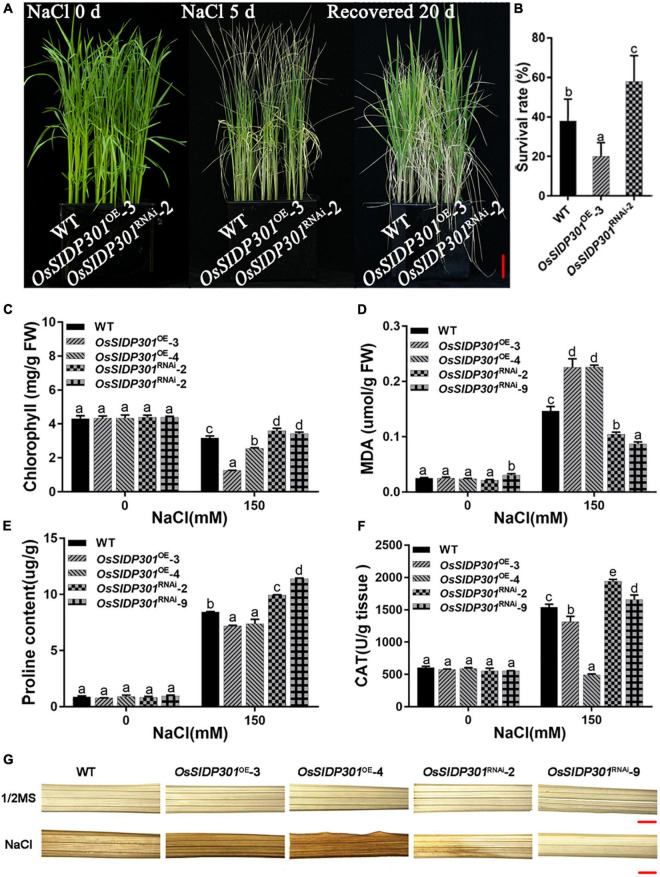
Physiological changes in *OsSIDP301* transgenic plants. **(A)** Phenotype of *OsSIDP301* transgenic plants that were treated with 150 mM NaCl solution for 5 days and then recovered for 20 days, bar = 3 cm. **(B)** Survival rate of the panel **(A)**. **(C)** The chlorophyll content of 3-week-old plant leaves with or without 150 mM NaCl treatment. **(D)** The MDA content of 3-week-old plant leaves with or without 150 mM NaCl treatment. **(E)** The proline content of 3-week-old plant leaves with or without 150 mM NaCl treatment. **(F)** The CAT activity of 3-week-old plant leaves with or without 150 mM NaCl treatment. **(G)** The DAB staining of 3-week-old plant leaves with or without 150 mM NaCl treatment, bar = 1 mm. Data are shown as mean ± SD (*n* = 3), different letters suggested significant differences at *P* < 0.05.

Thereafter, salt-stress response analysis was performed during different growth periods. For the germination stage (Figure 2D), the seeds were germinated in 1/2 MS medium with or without NaCl for 7 days. There was no difference between the WT and *OsSIDP301* transgenic plants grown in 1/2 MS medium (Figure 2E). When treated with NaCl, although there was no obvious difference between the WT and *OsSIDP301*^RNAi^ plants, it was significantly decreased in the *OsSIDP301*^OE^ plants compared with the WT plants (Figures 2F,G). For the seedling stage (Figure 2H), the seeds of all plants were germinated in 1/2 MS medium and then transferred to the medium with or without NaCl for continuous 7 days of treatment. There was no substantial difference between the WT and *OsSIDP301* plants grown in 1/2 MS medium. However, *OsSIDP301*^RNAi^ lines showed higher salt tolerance, with shoot length (+4.29%, +4.96%), root length (+14.27%, +18.26%), fresh weight (+7.48%, +9.2%), and dry weight (+7.79%, +16.23%) remarkably higher than that of the WT with NaCl treatment (Figures 2I–L). In contrast, *OsSIDP301*^OE^ plants were more sensitive than WT plants, with shoot length (–3.54%, –0.67%), root length (–2.01%, –8.96%), fresh weight (–1.29%, –0.57%), and dry weight (–6.29%, –15.98%) dramatically reduced compared to WT plants (Figures 2I–L). In addition, 20-day-old WT and *OsSIDP301* plants were treated with 150 mM NaCl solution for 5 days and then re-watered for 20 days. As expected, compared with WT, the survival rate of *OsSIDP301*^OE^ lines was significantly decreased but was elevated in *OsSIDP301*^RNAi^ (Figures 3A,B). These results indicate that *OsSIDP301* may act as a negative regulator of salt stress in rice.

**FIGURE 4:**
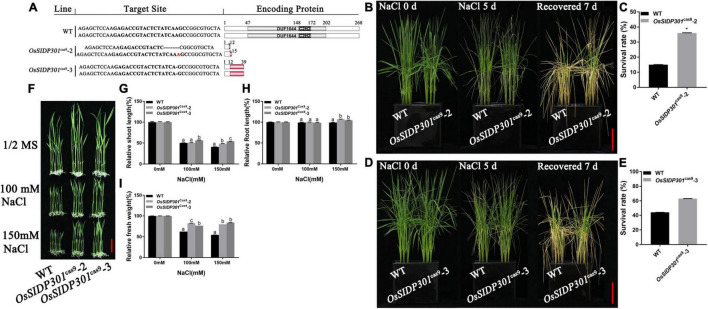
Mutants of *OsSIDP301* were conferred salt tolerance. **(A)** Mutation types of *OsSIDP301* mutants. **(B–E)** Phenotype and survival rate of mutants were treated with containing 150 mM NaCl solution for 5 days and then recovered for 7 days, bar = 4 cm. **(F)** Phenotypes of mutants with NaCl treatment at the seedling stage, bar = 2 cm. Comparisons of shoot length **(G)**, root length **(H)**, fresh weight **(I)** between WT and mutants with or without NaCl treatment for 7 days. Data are shown as mean ± SD (*n* = 8), different letters suggested significant difference at *P* < 0.05.

To further verify the salt tolerance phenotype, mutants were generated using the CRISPR/Cas9 genome-editing system. Two mutants of *OsSIDP301*, designated as *OsSIDP301*^cas9^-2 and *OsSIDP301*^cas9^-3, were chosen for further characterization (Figure 4A). The results showed that *OsSIDP301*^cas9^ plants displayed a similar tendency to *OsSIDP301*^RNAi^ lines when treated with NaCl. The 20-day-old *OsSIDP301*^cas9^ mutants were treated with 150 mM NaCl for 5 days and then re-watered for 7 days. Consequently, the survival rate of *OsSIDP301*^cas9^ lines was significantly increased compared with WT (Figures 4B–E). In addition, 3-day-old seedlings of the WT and *OsSIDP301*^cas9^ lines were grown with or without NaCl treatment for 7 days, respectively (Figure 4F). *OsSIDP301*^cas9^ lines showed higher salt tolerance than the corresponding WT, with an increase in shoot length, root length, and fresh weight (Figures 4G–I). In addition, there was also no significant difference at the germination stage between *OsSIDP301*^cas9^ and WT plants with or without NaCl treatment (Supplementary Figures 1A–C).

### Knockdown of *OsSIDP301* Reduces the ROS Levels in Rice Under Salt Stress

To further verify the effect of *OsSIDP301*, physiological parameters (including the total chlorophyll content, malondialdehyde [MDA], proline content, and CAT activity) were measured. The total chlorophyll content, which represents the presence of chlorosis, was less downregulated in *OsSIDP301*^RNAi^ lines than that of WT, whereas it was severely decreased in *OsSIDP301*^OE^ lines compared with WT under saline conditions (Figure 3C). MDA, which induces lipid peroxidation and represents cell oxidative damage, was markedly higher in *OsSIDP301*^OE^ lines but lower in *OsSIDP301*^RNAi^ lines than in the WT plants under salt stress (Figure 3D). In addition, proline and CAT, which play an essential role in protecting cells against oxidative stress caused by ROS, were increased in *OsSIDP301*^RNAi^ lines and decreased in *OsSIDP301*^OE^ lines compared to that of WT plants with salt treatment (Figures 3E,F). Moreover, 3,3′-diaminobenzidine (DAB) staining assay was used to determine the H_2_O_2_ content, which reflects the ROS levels. In comparison with WT leaves, *OsSIDP301*^OE^ leaves showed stronger staining, whereas only weak staining was observed in *OsSIDP301*^RNAi^ lines (Figure 3G). Taken together, these results suggested that *OsSIDP301* is crucial for maintaining cellular redox homeostasis.

### Knockdown of *OsSIDP301* Reduces the Na^+^ Content in Rice Under Salt Stress

**FIGURE 5:**
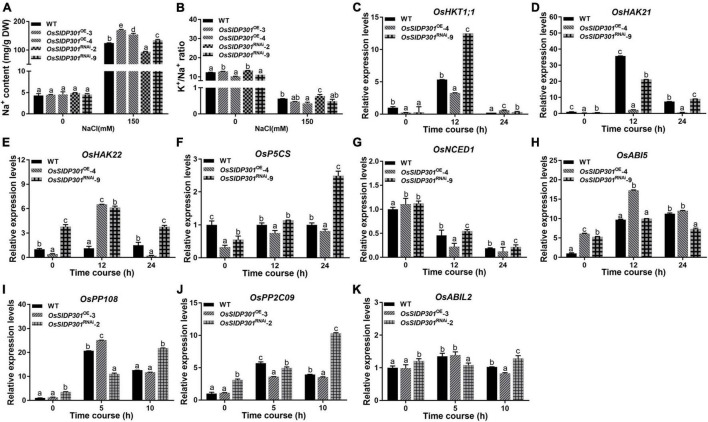
The expression levels of salt stress and ABA-related genes. The Na^+^ content **(A)** and K^+^/Na^+^ ratio **(B)** of 3-week-old plant leave with or without 150 mM NaCl treatment. **(C–H)** The expression level analysis of ion transporter *OsHKT1;1*, *OsHAK21*, *OsHAK22*, and salt-response genes *OsNCED1*, *OsP5CS*, and *OsABI5* with or without 150 mM NaCl treatment by using 2-week-old seedlings. **(I–K)** The expression level analysis of ABA signaling regulators *OsPP108*, *OsPP2C09*, and *OsABIL2* with 100 μM ABA treatment by using 2-week-old seedlings. Data are shown as mean ± SD (*n* = 3), different letters suggested significant differences at *P* < 0.05.

To examine whether the reduced growth and biomass of *OsSIDP301*^OE^ plants were also caused by osmotic stress, 3-day-old seedlings were planted with 0, 120, or 150 mM mannitol. Interestingly, there were no significant differences in *OsSIDP301* compared to the WT (Supplementary Figures 1D,E), indicating that ion toxicity rather than osmotic stress was the reason why *OsSIDP301*^OE^ plants exhibited reduced tolerance to salt stress. Subsequently, the Na^+^ content of the leaves of 4-week-old seedlings was measured in seedlings grown with and without NaCl treatment (Figures 5A,B). The results showed that Na^+^ content was significantly higher in the *OsSIDP301*^OE^ plants than in the WT plants, whereas it decreased in the *OsSIDP301*^RNAi^ plants with NaCl treatment. These data suggest that the enhanced salt tolerance in the *OsSIDP301*^RNAi^ lines may be due to reduced Na^+^ accumulation.

Additionally, we detected the expressions of salt ion transporters *OsHKT1;1* (Wang et al., 2015), *OsHAK21* (Shen et al., 2015), *OsHAK22* (He et al., 2019), and salt-response genes, including Δ′-*PYRROLINE-5-CARBOXYLATE SYNTHETASE* (P5CS; Zhang et al., 1995) and *9-CIS-EPOXYCAROTENOID DIOXYGENASE 1* (OsNCED1; Liu, 2017), which have been reported to positively regulate salt stress. *ABA INSENSITIVE 5* (*ABI5*) has been reported to negatively modulate salt stress (Zou et al., 2008). Our results showed that the expression levels of *OsHKT1;1*, *OsHAK21*, *OsHAK22*, *OsP5CS*, and *OsNCED1* were decreased, while *OsABI5* transcript was increased in *OsSIDP301*^OE^ plants (Figures 5C–H). However, an opposite trend was observed in *OsSIDP301*^RNAi^ plants. Taken together, these results demonstrate that *OsSIDP301* may enhance salt sensitivity by influencing the Na^+^ content and salt-response genes in rice.

### Transcriptome Analysis of *OsSIDP301*^OE^ and *OsSIDP301*^RNAi^ Plants

**FIGURE 6:**
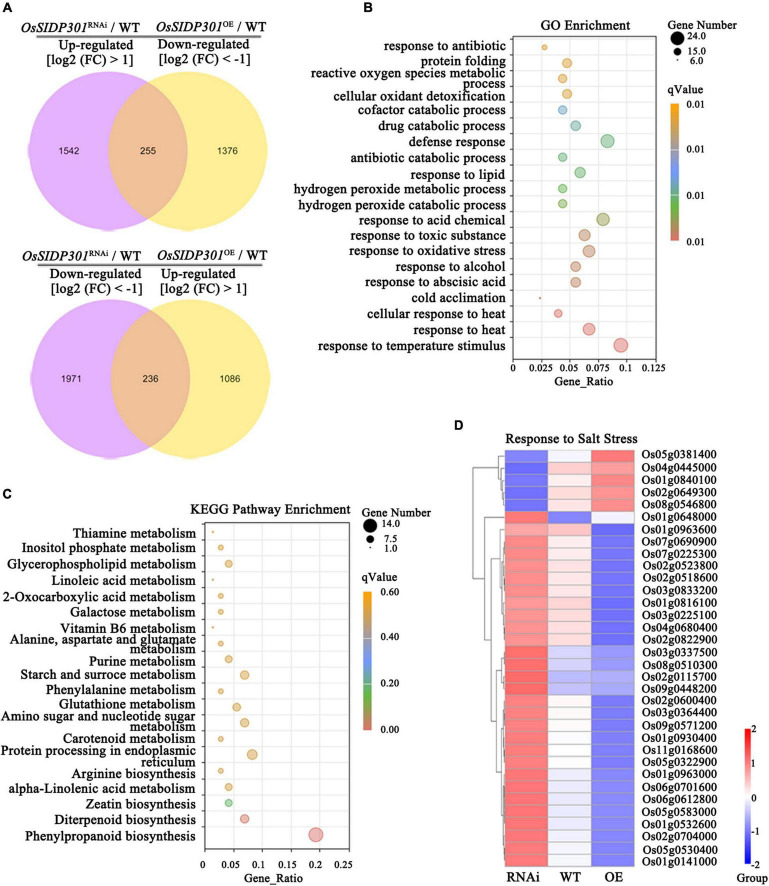
Transcriptome analysis of *OsSIDP301* transgenic plants. **(A)** Venn diagrams for the upregulated in *OsSIDP301^RNAi^* and downregulated in *OsSIDP301*^OE^ DEGs, and downregulated in *OsSIDP301*^RNAi^ and upregulated in *OsSIDP301*^OE^ DEGs with NaCl treatment. **(B)** Gene Ontology (GO) enrichment analysis of the **(A)** DEGs. **(C)** Kyoto Encyclopedia of Genes and Genomes (KEGG) enrichment analysis of the **(A)** DEGs. **(D)** Heatmaps showing the partially representative salt-related gene expression patterns between WT and transgenic plants under salt stress.

To elucidate the molecular mechanism of *OsSIDP301*, transcriptome deep sequencing (RNA-seq) was performed in the leaves of salt-stressed 3-week-old plants. With the threshold of significantly differentially expressed genes (DEGs) set at log_2_ (fold change) > 1 or log_2_ (fold change) < –1 and adjusted to *P* < 0.05, 4,002 DEGs (1,796 upregulated and 2,206 downregulated) were identified in *OsSIDP301*^RNAi^ compared with WT, and 2,951 DEGs (1,321 upregulated and 1,630 downregulated) in *OsSIDP301*^OE^ compared with WT under salt stress (Supplementary Figures 2A,B). We also identified that 255 DEGs were downregulated in *OsSIDP301*^OE^ and upregulated in *OsSIDP301*^RNAi^ plants, and 236 DEGs were upregulated in *OsSIDP301*^OE^ and downregulated in *OsSIDP301*^RNAi^ plants (Figure 6A). Gene Ontology (GO) enrichment analysis on these 491 DEGs revealed that genes were related to stress responses, and the GO term with the DEGs included “response to stress (heat, oxidative, antibiotic, etc.),” “protein folding,” “metabolic process,” and “catabolic process” (Figure 6B). Then, the Kyoto Encyclopedia of Genes and Genomes (KEGG) enrichment analysis showed that these DEGs were enriched in the “biosynthesis and metabolism” pathway that may be conducive to salt tolerance (Figure 6C). These salt-related DEGs encode diverse proteins, such as transcription factors (e.g., *Os05g0322900*, *Os02g0649300*), receptor-like kinases (e.g., *Os02g0822900*, *Os11g0168600*), antioxidant enzymes (e.g., *Os01g0963000*, *Os02g0115700*), and ion transporters (e.g., *Os04g0445000*, *Os01g0930400*) (Figure 6D).

As shown in Supplementary Figure 2, several representative genes from RNA-seq data were selected for confirmation using RT-qPCR. For example, *K*^+^-*efflux channels stelar K^+^ outward rectifier* was significantly upregulated in *OsSIDP301*^OE^ and downregulated in *OsSIDP301*^RNAi^ plants, but sodium transporter *OsHKT3*, Na^+^–K^+^ co-transport *OsHKT9*, and potassium transporters *OsHAK5* and *OsHAK17* were considerably upregulated in *OsSIDP301*^RNAi^ plants, whereas the opposite results were observed in *OsSIDP301^OE^* plants. The salt-related positive regulators, such as *RECEPTOR*-*LIKE CYTOPLASMIC KINASE 311*, *WRKY TRANSCRIPTION FACTOR 45*, and *C2 DOMAIN*-*CONTAINING PROTEIN* were significantly upregulated in *OsSIDP301*^RNAi^ plants. However, the salt-related negative regulators, such as *HOMEOBOX*-*LEUCINE ZIPPER PROTEIN HOX24*, *HEAT STRESS TRANSCRIPTION FACTOR B2b*, *PLASMA MEMBRANE PROTEIN 1*, and *MEDIATOR 37_1* were significantly upregulated in *OsSIDP301*^OE^ plants. These results were consistent with RNA-seq data and the role of *OsSIDP301* in salt-tolerance signaling, revealing that *OsSIDP301* acts as a negative regulator of salt stress.

### *OsSIDP301* Positively Regulates Abscisic Acid Signaling

**FIGURE 7:**
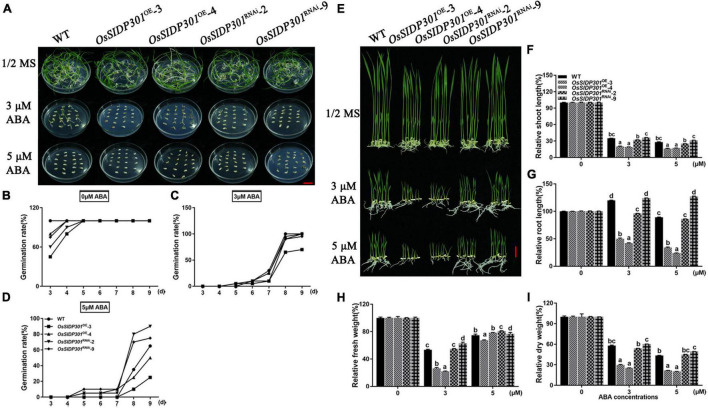
Phenotype of *OsSIDP301* transgenic plants with ABA treatment. **(A)** Phenotype of *OsSIDP301* transgenic plants with ABA treatment at germination stage, bar = 2 cm. Comparisons germination rate between WT, *OsSIDP301*^OE^, and *OsSIDP301*^RNAi^ plants with **(C,D)** or without ABA treatment **(B)**. **(E)** Phenotype of *OsSIDP301* transgenic plants with ABA treatment at the seedling stage, bar = 2 cm. Comparisons of shoot length **(F)**, root length **(G)**, fresh weight **(H)**, and dry weight **(I)** between WT, *OsSIDP301*^OE^, and *OsSIDP301*^RNAi^ plants with or without ABA treatment for 7 days. Data are shown as mean ± SD (*n* = 8), different letters suggested significant differences at *P* < 0.05.

As the expression of *OsSIDP301* was induced by ABA treatment, we hypothesized that *OsSIDP301* is associated with ABA signaling. To confirm this, the ABA sensitivity of *OsSIDP301* transgenic plants was first examined at the germination stage at different ABA concentrations (Figure 7A). The germination rate of *OsSIDP301*^OE^ lines was significantly lower than that of WT at 3 and 5 μM ABA treatments, whereas *OsSIDP301*^RNAi^ lines showed opposite results (Figures 7B–D). There were no significant differences between the transgenic and WT plants under normal conditions. For the seedling stage (Figure 7E), with different concentrations (3 and 5 μM) of ABA treatment for 7 days, the *OsSIDP301*^OE^ lines exhibited a severe decrease in shoot length (–15.1%, –11.9%), root length (–70.5%, –54.9%), fresh weight (–27.03%, –7.8%), and dry weight (–28.4%, –21.7%) compared with WT. In contrast, *OsSIDP301*^RNAi^ lines showed an obvious increase in shoot length (+0.9%, +2.8%), root length (+4.4%, +37.4%), fresh weight (+9.28%, +2.06%), and dry weight (+2.2%, +5.7%) after ABA treatment (Figures 7F–I).

In addition, we examined the expression of ABA signaling-related genes, including *PROTEIN PHOSPHATASE 2C 09* (*OsPP2C09*) (Miao et al., 2020), *PROTEIN PHOSPHATASE 108* (*OsPP108*) (Singh et al., 2015), and *OsABI-LIKE2* (*OsABIL2*) (Li et al., 2015), which negatively regulate ABA signaling. Their expression levels were upregulated in *OsSIDP301*^RNAi^, whereas slightly downregulated in *OsSIDP301*^OE^ lines compared to WT plants treated with 100 μM ABA (Figures 5I–K). Taken together, these results suggest that *OsSIDP301* plays a positive role in the ABA signaling pathway and participates in the ABA-mediated complex abiotic stress signal transduction pathway.

### *OsSIDP301* Negatively Regulates Grain Size

**FIGURE 8:**
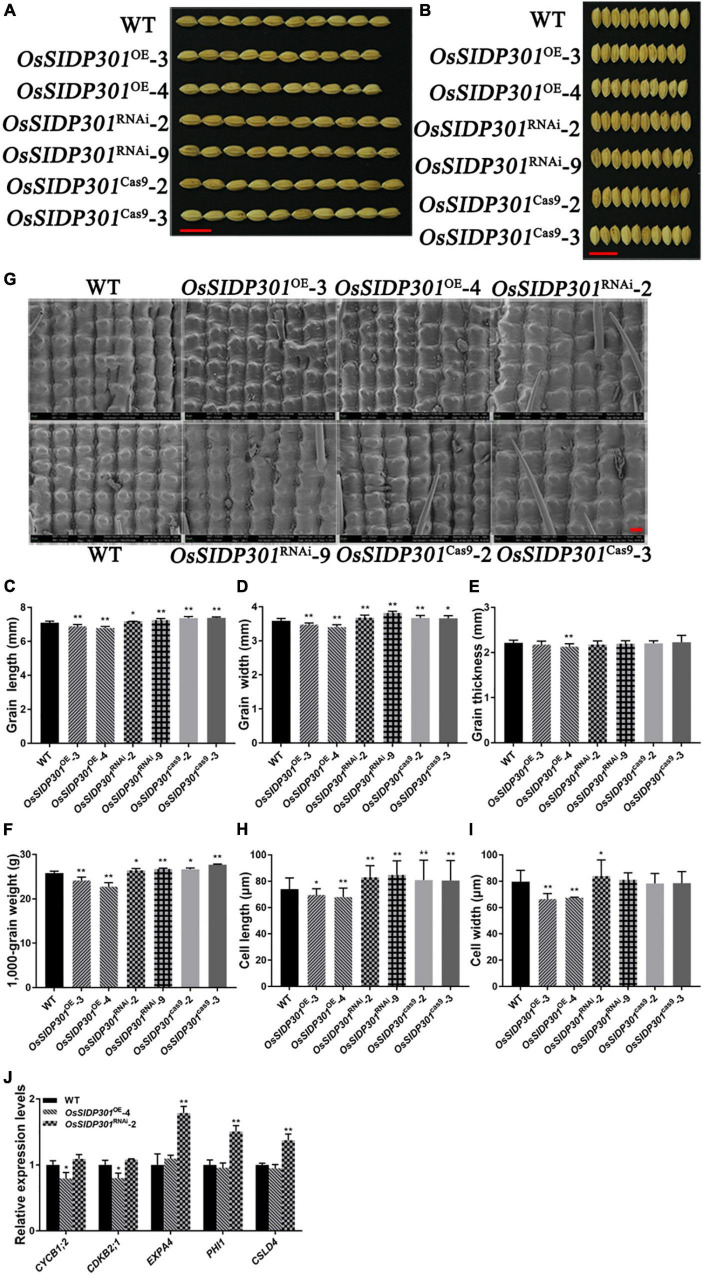
*OsSIDP301* regulates grain length and grain width. Morphology of grain length **(A)** and grain width **(B)** in WT, *OsSIDP301*^OE^, *OsSIDP301*^RNAi^ lines, and mutants, bar = 1 cm. Comparisons of grain length **(C)**, grain width **(D)**, grain thickness **(E)**, and 1,000-grain weight **(F)** between WT, *OsSIDP301*^OE^, *OsSIDP301*^RNAi^, and mutants (*n* = 100). **(G)** Scanning electron micrographs of the local outer surfaces of glumes in WT, *OsSIDP301*^OE^, *OsSIDP301*^RNAi^, and mutants, bar = 40 μm. Comparisons cell length **(H)** and cell width **(I)** between WT, *OsSIDP301*^OE^, *OsSIDP301*^RNAi^, and mutants on the surfaces of glumes. **(J)** Relative expression levels of cell cycle-related and cell expansion-related genes from RNA-seq analysis in WT and transgenic lines. Data are shown as mean ± SD (*n* = 3), Student’s *t*-test was used, *P < 0.05, **P < 0.01.

Since GUS staining showed that *OsSIDP301* was highly expressed in grains, it would be worthwhile to investigate whether *OsSIDP301* can function in grain development. As expected, the homozygous *OsSIDP301*^RNAi^ lines markedly increased in grain length (+1.13%, +3.8%), grain width (+2.8%, +5%), and 1,000-grain weight (+2.4%, +3.7%) (Figures 8A–D,F and Supplementary Figures 3A–E). Moreover, the number of secondary branches in the *OsSIDP301*^RNAi^ plants’ panicle increased compared to the WT (Supplementary Figures 3K,N). However, the homozygous *OsSIDP301*^OE^ lines showed opposite phenotypes. *OsSIDP301*^OE^ plants displayed decreased grain length (–2.96%, –4.5%), grain width (–3.1%, –5%), and 1,000-grain weight (–6%, –11.7%) (Figures 8A–D,F and Supplementary Figures 3A–E). Additionally, panicle length and the number of secondary branches in *OsSIDP301*^OE^ lines were significantly decreased than those in the WT plants (Supplementary Figures 3K–N). Furthermore, our results showed that the mutants displayed a significant increase in grain length (+3.8%, +3.9%), grain width (+2.5%, +2.2%), and 1,000-grain weight (+3.3%, +7.4%) compared to the WT (Figures 8C,D,F), but no obvious changes in grain thickness and panicle length were observed (Figure 8E and Supplementary Figures 3F–N). Together, these results indicate that *OsSIDP301* negatively regulates grain size and weight.

### *OsSIDP301* Regulates Grain Size by Promoting Cell Expansion

Cell proliferation and expansion are major factors that influence grain development. As the grain length of *OsSIDP301*^cas9^ mutants and *OsSIDP301*^RNAi^ plants increased, scanning electron microscopy (SEM) and paraffin section assays were used to observe the cells in spikelet hulls (Figure 8G and Supplementary Figures 4A,B). Compared with those of the WT, the epidermal cells of the glume were smaller in *OsSIDP301*^OE^ lines and enlarged in *OsSIDP301*^RNAi^ lines (Supplementary Figure 4B). Moreover, SEM results showed that the cell length of the glume was significantly increased in *OsSIDP301*^RNAi^ and *OsSIDP301*^cas9^ lines compared to the WT, but no significant difference in cell width was observed between *OsSIDP301*^cas9^ mutants and WT (Figures 8H,I). However, the cell length and width of the glume were significantly decreased in *OsSIDP301*^OE^ lines than those in WT. These results suggest that *OsSIDP301* negatively regulates grain size by influencing cell expansion. To explore the possible molecular pathway of *OsSIDP301* in the regulation of seed development, RNA-seq was performed in the young panicle of WT and *OsSIDP301* lines. A total of 514 differentially expressed genes (DEGs) were identified (Supplementary Figure 4C). GO enrichment and KEGG analysis showed that these DEGs were involved in biosynthesis and metabolism pathways (Supplementary Figures 4D,E). Among them, cell-expansion related genes, including *ALPHA-EXPANSIN 4* (*EXPA4*) (Choi et al., 2003), *PHOSPHATE-INDUCED PROTEIN 1* (Aya et al., 2014), and the cell-cycle related gene *CELLULOSE SYNTHASE-LIKE* (Yoshikawa et al., 2013), were significantly upregulated in *OsSIDP301*^RNAi^ lines compared to that in WT and *OsSIDP301*^OE^ plants (Figure 8J), indicating that the increased cell size in *OsSIDP301*^RNAi^ may result from the upregulated expression of genes that promote cell expansion. Taken together, these results indicate that *OsSIDP301* negatively regulates grain size by altering glume cell expansion.

### OsSIDP301 Interacts With OsBC1

**FIGURE 9:**
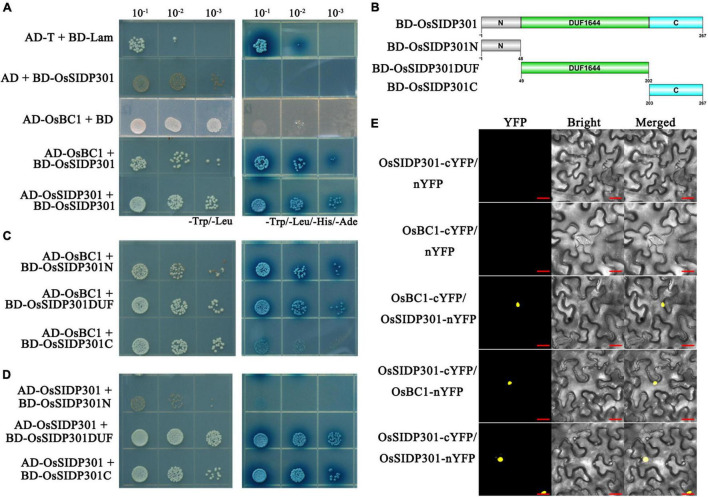
OsSIDP301 interacts with OsBC1. Yeast two-hybrid assay demonstrated the OsSIDP301 interacts with OsBC1 **(C)** and itself **(D)** by the full length **(A)**, N terminal, the DUF1644 domain, and the C terminal of OsSIDP301, respectively. **(B)** The protein truncation sites of OsSIDP301. **(E)** BiFC assay demonstrated the OsSIDP301 interacts with OsBC1 *in vivo*, the YFP fluorescence signals located in nuclear of *N. benthamiana* leaves, bar = 20 μm.

A yeast two-hybrid assay (Y2H) was used to screen for OsSIDP301-interacting proteins, with the OsSIDP301 protein used as a bait. *OsBC1*, which encodes a basic helix–loop–helix transcription activator and has been reported to positively regulate grain size (Jang et al., 2017), was identified as one of the interactive proteins (Figure 9A). Overexpression of *BC1* increases the grain size by promoting the expression of cell expansion-related genes, including *ALPHA-EXPANSIN 1* (*EXPA1*), *ALPHA-EXPANSIN 2* (*EXPA2*), *ALPHA-EXPANSIN 3* (*EXPA*3), and *EXPA*4 (Tanaka et al., 2009; Jang et al., 2017). In addition, we observed that OsSIDP301 could interact with itself, suggesting that OsSIDP301 might exert its function by forming dimers (Figure 9A). Subsequently, we cloned three types of truncations of full-length *OsSIDP301* based on the DUF1644 domain, including OsSIDP301N, OsSIDP301DUF, and OsSIDP301C, as shown in Figure 9B. The Y2H results showed that all truncations of OsSIDP301 could interact with OsBC1 and OsSIDP301, respectively (Figures 9C,D). BiFC assays were performed to further confirm the interaction between OsSIDP301 and OsBC1 *in vivo*. OsSIDP301 and OsBC1 were fused to the N-terminal (nYFP) and C-terminal (cYFP) of YFP, respectively. Confocal microscopy showed strong YFP fluorescence in the nucleus of *N. benthamiana* cells with OsBC1-cYFP and OsSIDP301-nYFP, OsSIDP301-cYFP and OsBC1-nYFP, or OsSIDP301-cYFP and OsSIDP301-nYFP, but not in the negative controls (Figure 9E). Taken together, these results indicate that OsSIDP301 could interact with OsBC1 and form homo-dimers in rice.

## Discussion

Several studies have reported that the DUF640 family is involved in grain development in rice (Li et al., 2012; Yan et al., 2013); the DUF966 family has been shown to respond to abiotic stress in rice (Luo et al., 2014); and the DUF1644 family is responsible for abiotic stress in rice and affects crop yield in maize (Guo et al., 2016; Li et al., 2016; Chen et al., 2022). The DUF1644 family is a plant-specific protein, and there are nine DUF1644 homologous genes in rice, while the functions of only two members have been identified and that of the others are still unclear. In this study, we provide evidence that the DUF1644 protein OsSIDP301 negatively regulates grain size and salt tolerance in rice.

### *OsSIDP301* Negatively Regulates Salt Tolerance

The major staple crops for eating are glycophytes, which are unable to complete their life cycle when salt concentrations in soil exceed 200 mM compared to halophytes (Munns and Tester, 2008; Flowers et al., 2015). Therefore, improving abiotic tolerance is essential for global food security and productivity. *OsSIDP361* and *OsSIDP366*, which encode the DUF1644 proteins with a conserved DUF1644 domain and zinc finger domain, respectively, have been reported to be associated with abiotic stress (Guo et al., 2016; Li et al., 2016). In this study, the overexpression of *OsSIDP301* was observed in response to salt stress (Figure 2A). Moreover, salt stress simulation at the germination and seedling stages showed that *OsSIDP301* might have a negative effect on salt tolerance (Figures 2, 3, 4). These results suggested that *OsSIDP301* plays an important role in abiotic stress tolerance. Previous studies have shown that salt stress induces the accumulation of MDA, proline, ROS, and Na^+^, as well as high activities of superoxide dismutase, peroxidase, and CAT, leading to disruption of cellular homeostasis and threatening plant development (Zhang et al., 2013; Kaur and Asthir, 2015; Liang et al., 2018). In this study, proline content was decreased in *OsSIDP301*^OE^ lines, whereas MDA content was increased with NaCl treatment; DAB staining also suggested that H_2_O_2_ content in *OsSIDP301*^OE^ lines was higher than that of the WT, which was consistent with inhibited CAT activity (Figure 3); and *OsSIDP301*^OE^ plants had a higher level of Na^+^ content when compared with WT under NaCl treatment (Figure 5A). The HKT, HAK, and AKT family members and salt-response genes have been reported to be related to the K^+^/Na^+^ balance in cells. For example, *OsHKT1;1* (*HKT4*) encodes a high-affinity potassium transporter that plays an essential role in controlling Na^+^ content and inhibiting Na^+^ toxicity in leaves, leading to enhanced salt tolerance in rice (Wang et al., 2015); *OsHAK21* encodes a potassium transporter and its mutants exhibit hypersensitivity to salt stress (He et al., 2019); and *OsABI5* encodes a bZIP transcription factor that negatively regulates salt tolerance *via* an ABA-dependent pathway (Zou et al., 2008). In this study, the expressions of *OsHKT1;1*, *OsHAK21*, *OsHAK22*, *OsABI5*, and *OsNCED1* were consistent with salt tolerance of *OsSIDP301* with NaCl treatment (Figures 5C–G). *P5Cs* has been reported to participate in proline biosynthesis (Zhang et al., 1995), and the overexpression of *P5Cs* in *OsSIDP301*^RNAi^ plants (Figure 5H) was consistent with higher proline content compared to WT (Figure 3E). In addition, RNA-seq data and RT-qPCR results confirmed that many positive factors were upregulated in *OsSIDP301*^RNAi^ plants (Figure 6 and Supplementary Figure 2), suggesting that *OsSIDP301* is involved in salt tolerance by regulating salt-related gene expression. Put together, these findings reveal that *OsSIDP301* negatively regulates salt tolerance by changing physiological parameters and participating in abiotic stress signaling pathways in rice.

### *OsSIDP301* Responds to Abscisic Acid Signaling

Abiotic stress-resistance systems are complex networks that include signal transduction, phytohormones, and functional gene regulation. ABA has been widely reported to respond to abiotic stress (Zhu, 2002). In this study, an obvious hypersensitive phenotype to ABA treatment was exhibited in *OsSIDP301*^OE^ plants at the seed germination and seedling growth stages (Figure 7), which was contrary to previous results on the relationship between ABA and stress resistance. However, *Oshox22* (Zhang et al., 2012), *bZIP TRANSCRIPTION FACTOR 05* (Tong et al., 2021), *OsABI5* (Zou et al., 2008), and *SlbZIP38* (Pan et al., 2017) have been found to negatively regulate salt or drought stress, but positively regulate ABA responses. In addition, *WRKY GENE 20* (Luo et al., 2013) and *OsbZIP71* (Liu et al., 2014) have been reported to positively regulate abiotic stress, but negatively regulate ABA responses. In this study, the overexpression of negative regulators in ABA signaling were observed in *OsSIDP301*^RNAi^ lines with ABA treatment, including *OsPP2C09* (Miao et al., 2020), *OsPP108* (Singh et al., 2015), and *OsABIL2* (Li et al., 2015). These results demonstrated that *OsSIDP301* is involved in the salt stress response through an ABA-dependent pathway.

### *OsSIDP301* Is Associated With Yield-Related Traits in Rice

Grain size (grain length and width)/weight is an important agronomic trait for crop production (Xing and Zhang, 2010). In rice, grain shape is related to cell expansion or proliferation. *SMALL GRAIN 11*, a new allele of *DWARF2*, positively regulates grain size and weight by promoting cell expansion in rice (Fang et al., 2016); *MEI2-LIKE PROTEIN 4* negatively regulates grain length and weight by controlling cell expansion in rice (Lyu et al., 2020); and *GRAIN WIDTH 6* enhances grain size and weight by promoting cell expansion (Shi et al., 2020). In this study, intense GUS staining and grain shape were observed in the seeds of transgenic plants, which showed that *OsSIDP301* plays an important role in grain development. Moreover, SEM and paraffin section results suggested that the smaller grain shape in *OsSIDP301*^OE^ lines might be caused by decreasing cell expansion (Figure 8 and Supplementary Figures 4A,B). BR has been reported to be associated with regulating grain shape (Li et al., 2019). *DWARF 61* encodes a BR receptor that positively regulates grain size by regulating downstream genes, and the overexpression of *BU1* showed typical BR phenotypes and larger grain size (Tanaka et al., 2009); *BC1* increased grain size by upregulating cell expansion-related genes, including *EXPA1*, *EXPA2*, *EXPA*3, and *EXPA*4 (Tanaka et al., 2009; Jang et al., 2017). In this study, OsSIDP301 was found to interact with OsBC1, and overexpression of *EXPA4* was observed in *OsSIDP301*^RNAi^ lines (Figures 9, 8J). These results suggest that *OsSIDP301* negatively regulates grain size by controlling cell expansion.

**FIGURE 10:**
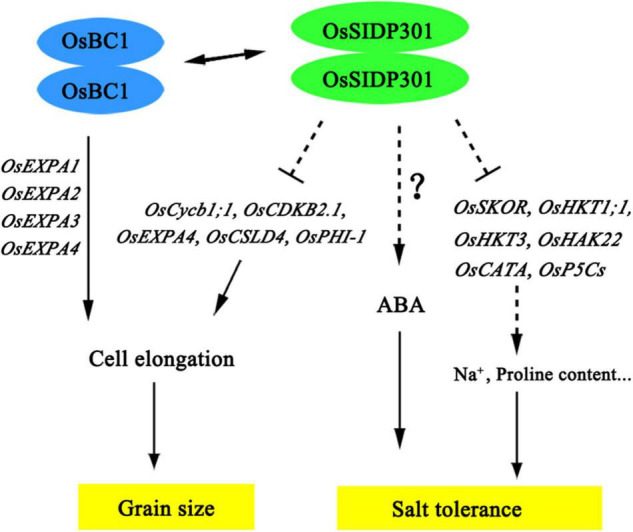
The proposed working model of *OsSIDP301* regulates salt tolerance and grain size. The dotted lines indicated indirect regulation, the two-way arrows indicate interaction of protein, and the solid lines indicate direct regulation.

In conclusion, our findings indicate that *OsSIDP301* plays a negative role in salt tolerance by regulating salt-related genes and negatively regulating grain size and weight by promoting cell expansion in spikelet hulls. Moreover, OsSIDP301 interacts with OsBC1, which also plays an important role in regulating grain size (Figure 10). Taken together, the knockdown of *OsSIDP301* can enhance grain size/weight and salt tolerance in rice. These findings of the functional characteristics of *OsSIDP301* complement the mechanism of yield and resistance synergy, which could provide a new target for cultivating high-yield and stress-resistant varieties in rice.

## Data Availability Statement

The datasets presented in this study can be found in online repositories. The names of the repository/repositories and accession number can be found below: NCBI SRA, PRJNA852225, and PRJNA852226.

## Author Contributions

LC and YC supervised the project and revised the article. LC, YC, and LG designed the experiments. LG performed most of the experiments, analyzed the data, and wrote the article. YC, HG, and CG helped with manuscript revision. XL conducted the screening of some transgenic plants and yeast. HB, MT, LH, and YY assisted in the experiments. All authors contributed to the article and approved the submitted version.

## Conflict of Interest

The authors declare that the research was conducted in the absence of any commercial or financial relationships that could be construed as a potential conflict of sdainterest.

## Publisher’s Note

All claims expressed in this article are solely those of the authors and do not necessarily represent those of their affiliated organizations, or those of the publisher, the editors and the reviewers. Any product that may be evaluated in this article, or claim that may be made by its manufacturer, is not guaranteed or endorsed by the publisher.

## Footnotes

1
https://rapdb.dna.affrc.go.jp/

2
www.graphpad.com/

## Supplementary Material

Supplementary Figure 1 The germination rate with NaCl treatment in mutants and osmotic stress for *OsSIDP301* lines. **(A)** Phenotype of mutants at germination stage with or without NaCl treatment, bar = 2 cm. Comparisons germination rate between WT and mutants with or without NaCl treatment for 2 days **(B)** and 3 days **(C)**, data was shown as mean ± SD (*n* = 8). **(D)** Phenotype of *OsSIDP301* transgenic plants at the seedling stage with or without Mannitol treatment, bar = 2 cm. **(E)** Comparisons of shoot length between WT, *OsSIDP301*^OE^, and *OsSIDP301*^RNAi^ plants for 7 days with Mannitol treatment, data was showed as mean ± SD (*n* = 7).

Supplementary Figure 2 **(A, B)** Volcano plots comparing the transcriptomes between *OsSIDP301*^RNAi^ and *OsSIDP301*^OE^ with the WT. The green and red dots represent downregulated DEGs with log_2_(FC) < -1 and upregulated DEGs with log_2_(FC) > 1, respectively. The blue dots represent no significant difference in transcriptomes. **(C–V)** Relative expression levels of DEGs from RNA-seq by RT-qPCR analysis. Data were shown as mean ± SD (*n* = 3).

Supplementary Figure 3 Increased *OsSIDP301* expression caused smaller grains and shorter panicles. **(A)** Morphology of grain length and grain width in WT, *OsSIDP301*^OE^, and *OsSIDP301*^RNAi^ lines. Comparisons of grain length **(B)**, grain width **(C)**, grain thickness **(D)**, and 1,000-grain weight **(E)** between WT, *OsSIDP301*^OE^ and *OsSIDP301*^RNAi^ lines (*n* = 100). **(F)** Morphology of grain length and grain width in WT and mutants. Comparisons of grain length **(G)**, grain width **(H)**, grain thickness **(I)**, and 1,000-grain weight **(J)** between WT and mutants. **(K)** Morphology of panicle length in WT, *OsSIDP301*^OE^, *OsSIDP301*^RNAi^ lines, and mutants, bar = 3 cm. Comparisons of panicle length **(M)**, primary branches **(L)**, and secondary branches **(N)** between WT, *OsSIDP301*^OE^, *OsSIDP301*^RNAi^ lines, and mutants (*n* = 4). Data were shown as mean ± SD, Student’s *t*-test was used, **P* < 0.05, ^**^*P* < 0.01.

Supplementary Figure 4 Cytological analysis of spikelet before fertilization and transcriptome analysis between WT and *OsSIDP301* plants in panicle. **(A)** Longitudinal sections of spikelet before fertilization, bar = 1 mm. **(B)** The local longitudinal sections of spikelet regions that were indicated by a red rectangle on panel **(A)** were enlarged 20-fold, the local cell length was indicated by the red line, bar = 250 μm. **(C)** Venn diagrams of the DEGs between WT and *OsSIDP301* plants in panicle. **(D)** GO enrichment analysis of all the **(C)** DEGs. **(E)** KEGG enrichment of all the **(C)** DEGs.

Supplementary Table 1 Primers in this study.

Supplementary Table 2 Differentially Expressed Genes (DEGs) between WT, *OsSIDP301*^OE^, and *OsSIDP301*^RNAi^ from RNA-seq analysis, and using leaves as the material with 150 mM NaCl treatment for 3 days.

Supplementary Table 3 Differentially Expressed Genes (DEGs) between WT, *OsSIDP301*^OE^, and *OsSIDP301*^RNAi^ from RNA-seq analysis, and using young panicle as the material.
