# Supplementary figures and images for: OsSIDP301, a Member of the DUF1644 Family, Negatively Regulates Salt Stress and Grain Size in Rice
Source: Front Plant Sci. 2022 Jul 28;13:863233. doi: 10.3389/fpls.2022.863233 (PMC9366248; doi:10.3389/fpls.2022.863233)

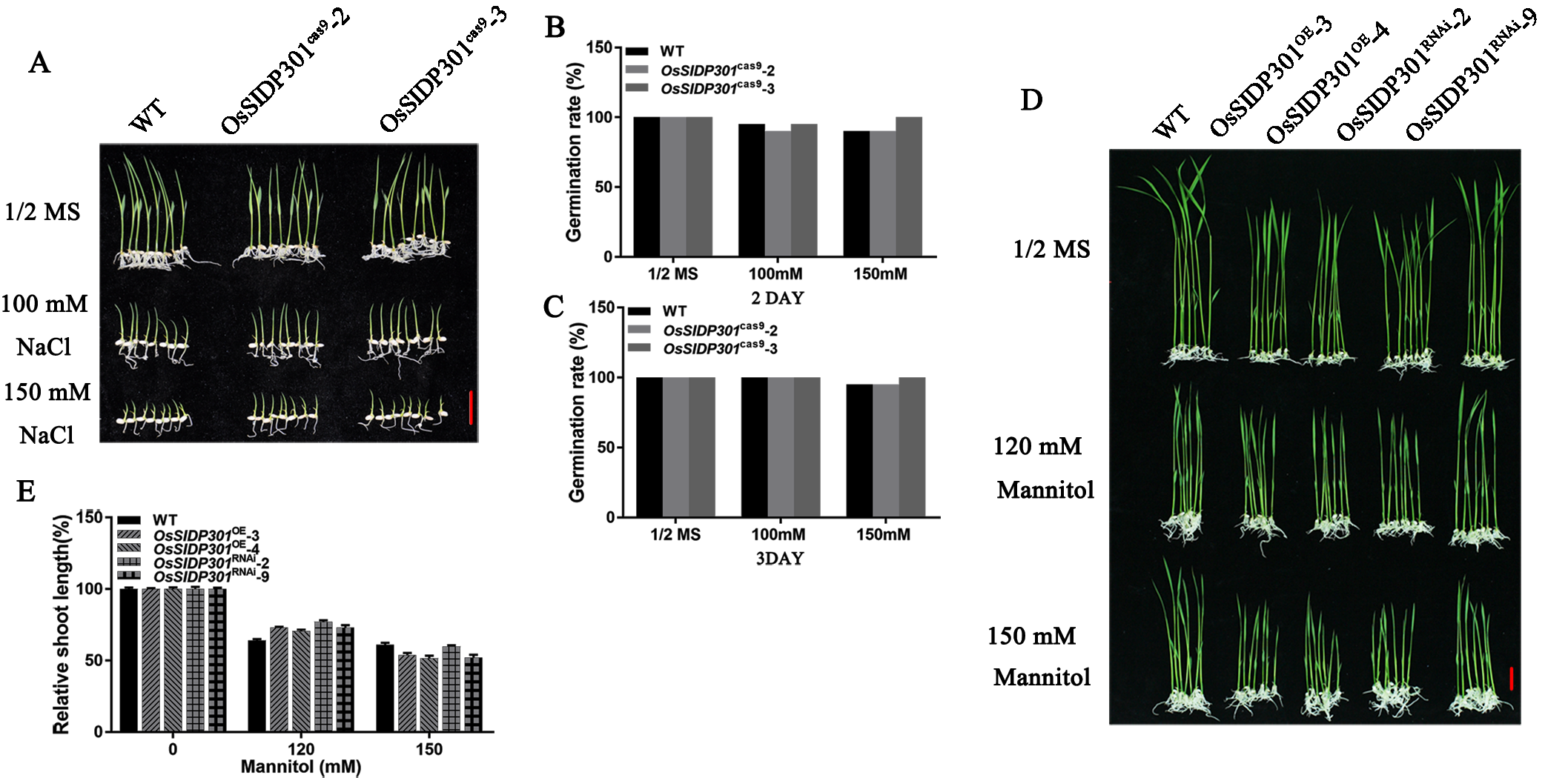

Supplement: Supplementary Figure 1 — The germination rate with NaCl treatment in mutants and osmotic stress for OsSIDP301 lines. (A) Phenotype of mutants at germination stage with or without NaCl treatment, bar = 2 cm. Comparisons germination rate between WT and mutants with or without NaCl treatment for 2 days (B) and 3 days (C), data was shown as mean ± SD (n = 8). (D) Phenotype of OsSIDP301 transgenic plants at the seedling stage with or without Mannitol treatment, bar = 2 cm. (E) Comparisons of shoot length between WT, OsSIDP301OE, and OsSIDP301RNAi plants for 7 days with Mannitol treatment, data was showed as mean ± SD (n = 7). [file Image_1.TIF]

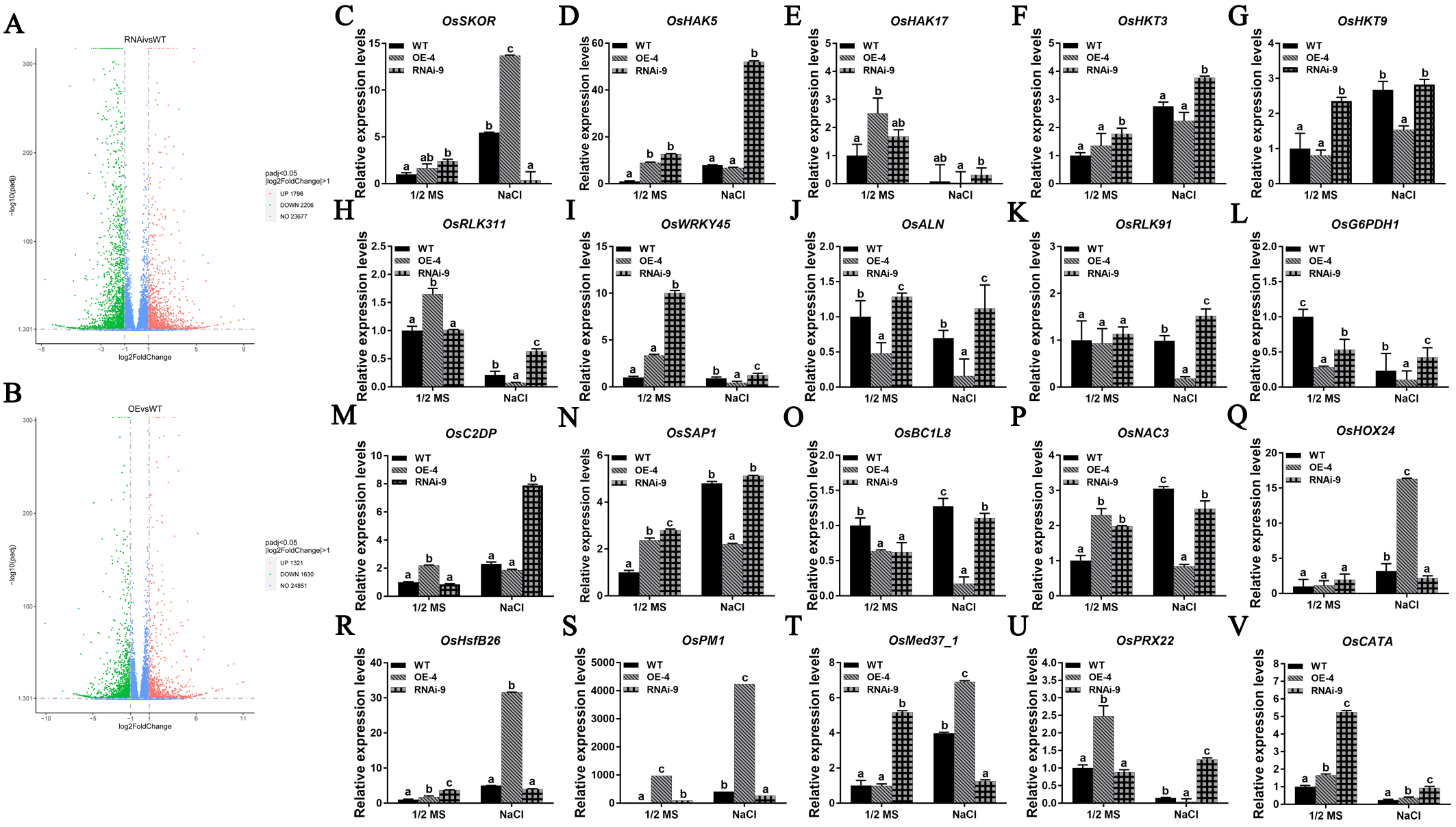

Supplement: Supplementary Figure 2 — (A, B) Volcano plots comparing the transcriptomes between OsSIDP301RNAi and OsSIDP301OE with the WT. The green and red dots represent downregulated DEGs with log2(FC) < -1 and upregulated DEGs with log2(FC) > 1, respectively. The blue dots represent no significant difference in transcriptomes. (C–V) Relative expression levels of DEGs from RNA-seq by RT-qPCR analysis. Data were shown as mean ± SD (n = 3). [file Image_2.TIF]

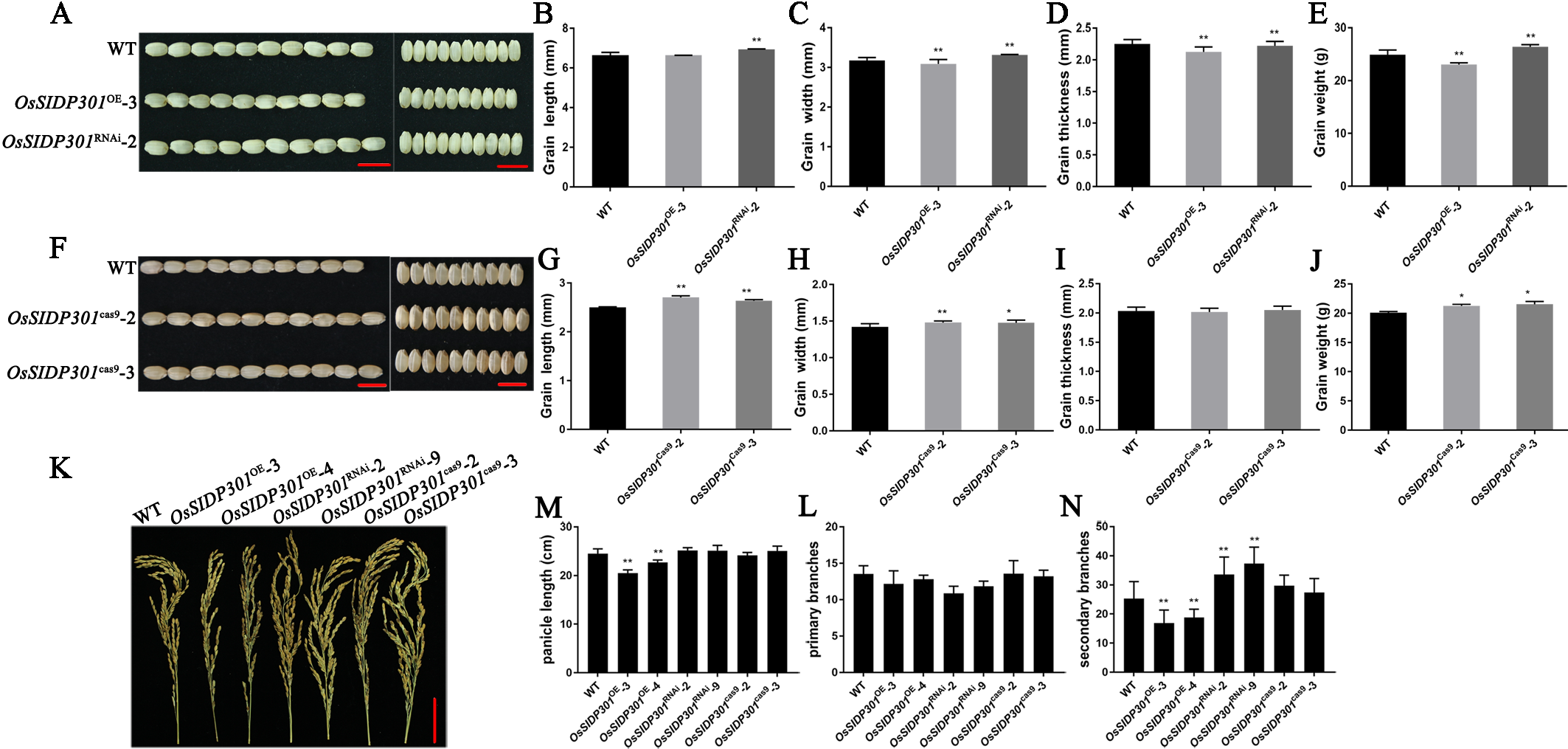

Supplement: Supplementary Figure 3 — Increased OsSIDP301 expression caused smaller grains and shorter panicles. (A) Morphology of grain length and grain width in WT, OsSIDP301OE, and OsSIDP301RNAi lines. Comparisons of grain length (B), grain width (C), grain thickness (D), and 1,000-grain weight (E) between WT, OsSIDP301OE and OsSIDP301RNAi lines (n = 100). (F) Morphology of grain length and grain width in WT and mutants. Comparisons of grain length (G), grain width (H), grain thickness (I), and 1,000-grain weight (J) between WT and mutants. (K) Morphology of panicle length in WT, OsSIDP301OE, OsSIDP301RNAi lines, and mutants, bar = 3 cm. Comparisons of panicle length (M), primary branches (L), and secondary branches (N) between WT, OsSIDP301OE, OsSIDP301RNAi lines, and mutants (n = 4). Data were shown as mean ± SD, Student’s t-test was used, *P < 0.05, **P < 0.01. [file Image_3.TIF]

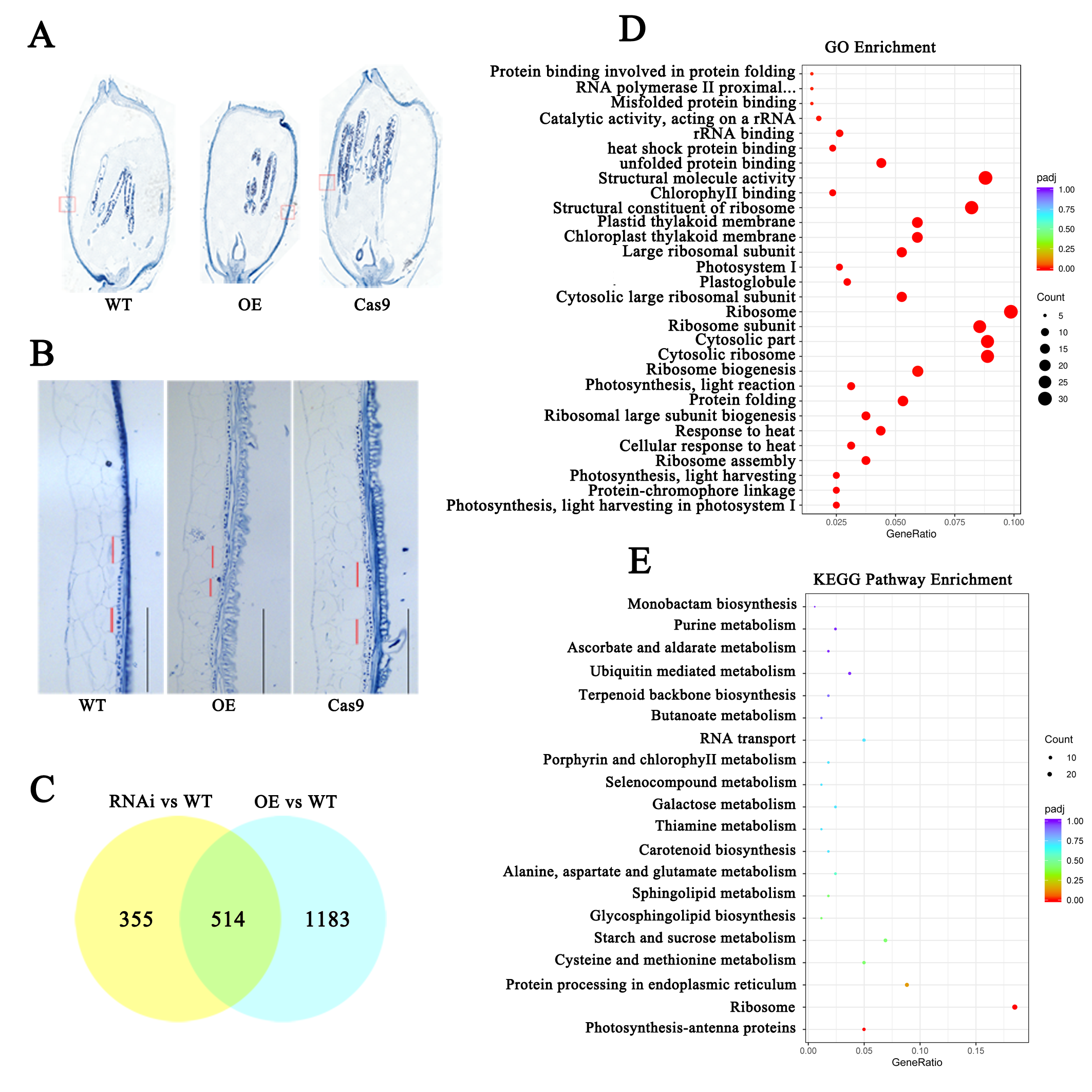

Supplement: Supplementary Figure 4 — Cytological analysis of spikelet before fertilization and transcriptome analysis between WT and OsSIDP301 plants in panicle. (A) Longitudinal sections of spikelet before fertilization, bar = 1 mm. (B) The local longitudinal sections of spikelet regions that were indicated by a red rectangle on panel (A) were enlarged 20-fold, the local cell length was indicated by the red line, bar = 250 μm. (C) Venn diagrams of the DEGs between WT and OsSIDP301 plants in panicle. (D) GO enrichment analysis of all the (C) DEGs. (E) KEGG enrichment of all the (C) DEGs. [file Image_4.TIF]
